# RTS,S malaria vaccine efficacy and immunogenicity during *Plasmodium falciparum* challenge is associated with HLA genotype

**DOI:** 10.1016/j.vaccine.2018.01.069

**Published:** 2018-03-14

**Authors:** C.M. Nielsen, J. Vekemans, M. Lievens, K.E. Kester, J.A. Regules, C.F. Ockenhouse

**Affiliations:** a London School of Hygiene and Tropical Medicine, London, UK; b Jenner Institute, University of Oxford, Oxford, UK^1^; c GSK Vaccines, Rixensart, Belgium; d Walter Reed Army Institute of Research, Silver Spring, MD, USA; e Sanofi Pasteur, Swiftwater, PA, USA^1^; f USAMRIID, Frederick, MD, USA^1^; g PATH Malaria Vaccine Initiative, Washington DC, USA

**Keywords:** Malaria, RTS,S, HLA, Antibody, Protection

## Abstract

Although RTS,S remains the most advanced malaria vaccine, the factors influencing differences in vaccine immunogenicity or efficacy between individuals or populations are still poorly characterised. The analyses of genetic determinants of immunogenicity have previously been restricted by relatively small sample sizes from individual trials. Here we combine data from six Phase II RTS,S trials and evaluate the relationship between HLA allele groups and RTS,S-mediated protection in controlled human malaria infections (CHMI), using multivariate logistic or linear regression. We observed significant associations between three allele groups (HLA-A^∗^01, HLA-B^∗^08, and HLA-DRB1^∗^15/^∗^16) and protection, while another three allele groups (HLA-A^∗^03, HLA-B^∗^53, and HLA-DRB1^∗^07) were significantly associated with lack of protection. It is noteworthy that these ‘protective’ allele groups are thought to be at a lower prevalence in sub-Saharan African populations than in the UK or USA where these Phase II trials occurred. Taken together, the analyses presented here give an indication that HLA genotype may influence RTS,S-mediated protective efficacy against malaria infection.

## 1 Introduction

Malaria caused by the *Plasmodium falciparum* parasite caused 438,000 deaths in 2015 and remains one of the major causes of mortality in children under five-years old in sub-Saharan Africa [1]. RTS,S is a candidate malaria vaccine antigen including a carboxy-terminal segment of the *P. falciparum* circumsporozoite (CS) protein fused to the hepatitis B surface antigen (HBsAg). When simultaneously expressed in yeast cells together with free HBsAg, these antigens assemble into particulate structures. The RTS,S antigen is formulated with the AS01E adjuvant system which contains the immunostimulants MPL, QS21, and liposomes. RTS,S/AS01 has been tested in a Phase III trial [2] and received a positive opinion from the European Medicines Agency [3], but further evaluation is ongoing and the underlying mechanisms of action are still not well understood [4]. Furthermore, there is significant variation in responses of vaccinated subjects both in the field and in controlled human malaria infections (CHMI) in non-endemic settings. It is of interest to understand what factors may contribute to this heterogeneity in order to guide further development and improvement of malaria vaccination strategies.

While there are many parameters that may contribute to vaccine responses, it is well-established that an individual subject’s genetics, such as HLA haplotype, can play a key role (reviewed in [5]). HLA alleles vary enormously at a population level and influence an individual’s response to a pathogen or vaccine antigen as the specificity of the peptide-binding pockets of each HLA molecule restricts the peptides that can be processed and presented to T cells. Associations between HLA background and immunogenicity have been demonstrated for a range of vaccines, including hepatitis B, influenza, anthrax, measles, rubella, and meningococcal C [5], [6], [7], [8]. It therefore seems likely that HLA may play a role in RTS,S responses, particularly given the strong evidence of associations between hepatitis B vaccine immunogenicity (as the RTS,S antigen includes CS protein co-expressed on the hepatitis B vaccine particle) and HLA-DR, HLA-DQ, and HLA-DP alleles (as reviewed in a 2013 meta-analysis [6], [9], [10].

While the protection mediated by RTS,S is thought to be primarily dependent on antibodies, CD4+ T cell responses have also been shown to correlate with protection in some studies (reviewed in [11]). As HLA Class II alleles will influence the CD4+ T cell response, HLA haplotypes could therefore potentially explain some of the heterogeneity in protective efficacy observed in CHMI. Indeed, one allele group associated with lower antibody titers to HBsAg, HLA-DRB1^∗^07, was detected in a significantly higher proportion of non-protected subjects as compared to protected subjects in a small CHMI trial (*n* = 26, Fisher’s exact test *p* = 0.03 [12]).

Additionally, although RTS,S clinical trial data have not demonstrated CD8+ T cell activation (reviewed in [11]), it is possible that evaluations of cellular immunity using peripheral blood are not representative of responses in the liver. The portion of CS protein expressed in RTS,S contains both CD4+ and CD8+ T cell epitopes [13] and CS protein is likely expressed early in the liver stage, which would allow HLA Class I presentation by hepatocytes to CD8+ T cells [13], [14].

Any evidence of an association between HLA haplotype and RTS,S vaccination outcome may contribute to a better understanding of mechanisms of vaccine action, and help explain the subject response heterogeneity in vaccine trials. The availability of pooled data from various Phase II trials permits a higher-powered analysis of HLA associations with protection and immunogenicity.

## 2 Materials and methods

### 2.1 Ethics statement

All studies and analyses have been performed under due IRB authorization and oversight, with subject informed consent.

### 2.2 Clinical trials included

Two hundred and twenty-two subjects vaccinated with RTS,S in six different CHMI clinical trials, and treated as per protocol, were available for inclusion in this analysis, as summarized in Table 1. Further details on the trials can be found using the clinicaltrials.gov identifier numbers and/or in the published reports of the main findings: WRMAL-003 [15], WRMAL-012 [16], MAL027 ([17]; NCT00075049), MAL068 ([18]; NCT01366534), MAL071 ([4]; NCT01857869), and VAC055 ([19]; NCT01883609). The main differences between these trials relate to the vaccination schedule and adjuvant used in the RTS,S formulation (see Table 1).

**Table 1:** Summary of subjects who received RTS,S and CHMI included in analyses.

Clinical Trial	Reference	Adjuvant	Schedule	Sample Size
WRMAL-003	[15]	AS02	RRR	19
WRMAL-012	[16]	AS02	RRR	20
MAL027	[17]	AS01 AS02	RRR RRR	26 33
MAL068	[18]	AS01 AS01	RRR ARR	21 25
MAL071	[4]	AS01 AS01	RRR RRr	16 30
VAC055	[19]	AS01 AS01	RRR RARR + MVA	15 17

**‘**Adjuvant’ indicates adjuvant included in the RTS,S formulation. ‘Schedule’ denotes the vaccination schedule: the standard 0/1/2 month RTS,S regimen (RRR); an adenovirus prime followed by two RTS,S doses after 1/2 months (ARR); the delayed fractional dose RTS,S regimen where the final dose was reduced and delayed to 7 months (RRr); or, the standard 0/1/2 month RTS,S regimen with adenovirus and modified vaccinia Ankara viral vectors against ME-TRAP at two weeks and 10 weeks, respectively (RARR + MVA).

### 2.3 HLA typing

HLA typing was performed on whole blood or buccal samples using standardized high resolution molecular typing (PCR) at the Georgetown University C. W Bill Young Marrow Donor Service and Testing Center, Washington DC; the Transplant Immunology Laboratory at the Walter Reed National Military Medical Center, Bethesda, MD, or the University of Oxford, Oxford, UK. For analysis, subjects were then classified based on broad HLA class I and class II related groups (equivalent to serologically defined antigens) without further stratification by 4-digit HLA allele definition. To note, the HLA-DRB1^∗^02 serotype consists of the HLA-DRB1^∗^15/^∗^16 allele groups, and thus for clarity we have used the designation HLA-DRB1^∗^15/^∗^16 throughout this paper.

### 2.4 Controlled human malaria infection (CHMI) model

Subjects included in these analyses underwent mosquito-delivered controlled human malaria infection (CHMI) according to comparable standard procedures [20] following vaccination to assess efficacy. This protocol was been previously described and is used to generate efficacy endpoint data in the trials included here [4], [15], [16], [17], [18], [19]. Briefly, vaccinees are infected with 3D7 strain *P. falciparum* through the bite of five infected mosquitoes, approximately three weeks after the final vaccination. Parasitemia is then monitored daily, using blood smears, between five and 18 days post-challenge, and then every alternate day until 28 days post-challenge. To note, unvaccinated subjects - who all became infected during CHMI - are not included in the analyses.

### 2.5 ELISAs

ELISAs for anti-CS protein (NANP repeat) IgG titres (henceforth referred to as anti-CS titres) were performed as previously described [4], [15], [16], [17], [18].

### 2.6 Statistics

Trial participants were coded 0/1 for each of the 40 alleles groups detected, and also assigned codes for adjuvant received, vaccination schedule, whether they received a viral vector in addition to RTS,S, and the trial in which they participated. All statistical analyses were performed in STATA.

#### 2.6.1 Primary analyses

Odds ratios (ORs) of protected subjects with a specific allele group versus protected subjects without the allele group were calculated using logistic regression. Kaplan-Meier curves were also produced to illustrate these unadjusted analyses. Logistic regression was then used to adjust for trial, adjuvant, and schedule (differentiating between standard schedule, delayed fractional dose, and schedules with an adenovirus viral vector or adenovirus and modified vaccinia Ankara [MVA] viral vectors). We do not consider adjusting for multiplicity of testing to be strictly necessary [21], as detailed in the Section Discussion.

#### 2.6.2 Secondary analyses

Supplementary to the efficacy analyses, immunogenicity analyses were also performed comparing the anti-CS titres between subjects with and without an allele group using linear regression, adjusting for trial or trial, adjuvant, and schedule. Primary analyses were unadjusted for other allele groups, however for allele groups where significant associations were detected with protection or immunogenicity, logistic regression models were re-run adjusting for other significant allele groups, or anti-CS titres at day of challenge.

## 3 Results

A total of 222 RTS,S-vaccinated subjects, with 40 different broad serotype HLA-A, HLA-B, and HLA-DRB1 allele groups, were available for analysis (Table 1). The prevalence of HLA-A/B/DRB1 allele groups varied across the six trials [4], [15], [16], [17], [18], [19], emphasizing the importance of adjusting for trial in the analyses, as described in Section Materials and Methods.

### 3.1 HLA allele groups are associated with protective efficacy of RTS,S vaccination

**Fig. 1:**
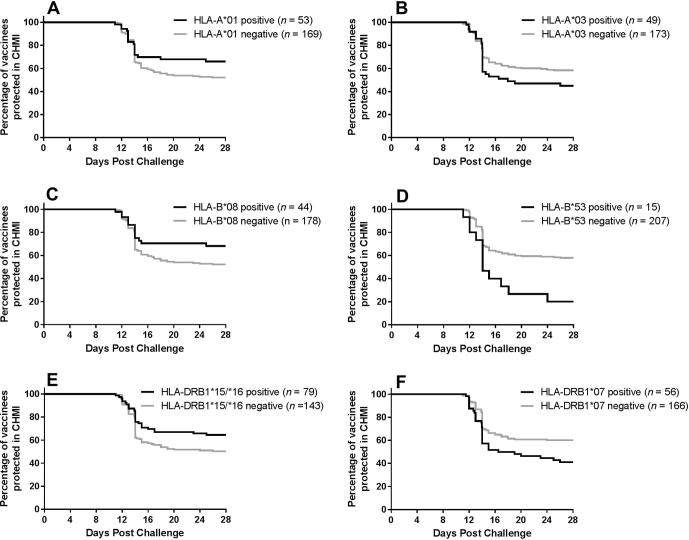
Allele groups significantly associated with protection in controlled human malaria infection (CHMI) are not associated with delays to parasitemia. The percentage of vaccinees who are protected in CHMI following vaccination (i.e. have not been diagnosed with malaria) is plotted against days since malaria challenge, comparing donors positive or negative for HLA-A*01 (A), HLA-A*03 (B), HLA-B*08 (C), HLA-B*53 (D), HLA-DRB1*15/*16 (E), and HLA-DRB1*07 (F) allele groups. The number of donors positive or negative for each allele group (*n*) out of a possible 222 subjects is given in the figure legends.

The proportion of subjects protected was compared between subjects positive and negative for each allele group using logistic regression, adjusting for trial, adjuvant and schedule. Three of the 40 allele groups had insufficient numbers of positive donors for analysis by logistic regression. Of the 37 allele groups analysed, six had statistically significant associations with CHMI outcome as summarised in Table 2 and Fig. 1.

**Table 2:** Association of HLA-A/B/DRB1 allele groups with protection in CHMI.

HLA	*n*	Odds Ratio	95% CI	*p* value
HLA-A*01	53	2.36	1.15–4.81	0.019
HLA-A*03	49	0.44	0.21–0.91	0.026
HLA-B*08	44	2.51	1.16–5.44	0.020
HLA-B*53	15	0.16	0.04–0.66	0.011
HLA-DRB1*15/*16	79	1.90	1.03–3.50	0.041
HLA-DRB1*07	56	0.36	0.18–0.73	0.005

HLA = broad serotype; *n* = number of subjects positive for allele group. Odds ratio, 95% confidence interval (CI), and *p* value refer to comparison of proportion of subjects protected between subjects positive and negative for an allele group using logistic regression and adjusting for trial, adjuvant, and schedule.

Three allele groups were associated with protection, HLA-A^∗^01 (OR: 2.36, *p* = 0.019), HLA-B^∗^08 (OR: 2.51, *p* = 0.020), and HLA-DRB1^∗^15/∗16 (OR: 1.90, *p* = 0.041), and three allele groups were associated with lack of protection HLA-A^∗^03 (OR: 0.44, *p* = 0.026), HLA-B^∗^53 (OR: 0.16, *p* = 0.011), and HLA-DRB1^∗^07 (OR: 0.36, *p* = 0.005). Interestingly, there was no strong evidence that protective HLA-A^∗^01, HLA-B^∗^08, and HLA-DRB1^∗^15/^∗^16 allele groups were associated with substantial delays to parasitemia (Fig. 1).

The results were very similar when analyses were adjusted for trial only, or for trial, adjuvant and schedule (Supplemental Table 1). Although individual trials do contain multiple adjuvant and schedule regimes (Table 1), this indicates that any variation in the relationship between HLA and RTS,S-mediated protection driven by adjuvant or schedule was likely adequately captured by controlling for trial alone. Similarly, if subjects who received viral vectors in addition to RTS,S were excluded from the analyses, the majority of the associations remained statistically significant at the 0.05 level, with the exception of HLA-A∗03. We interpret this to be a reflection of the decreased power due to the reduced sample size rather than an impact of the use of adenovirus or MVA viral vectors on the associations between HLA and protection (Supplemental Table 1), although it could be of interest to compare vaccine administration modalities in future studies.

### 3.2 HLA allele groups significantly associated with RTS,S-mediated protection may not be independent

We next acknowledged the probability of linkage disequilibrium and considered whether the associations of these six allele groups were independent of each other by repeating the logistic regression analyses, adjusting for the other allele groups. Using this approach, a statistically significant association between HLA-DRB1^∗^15/^∗^16 and protection was still detected, indicating that a putative protective effect mediated by this allele group is not linked to the presence of HLA-A^∗^01 or HLA-B^∗^08. Similarly, the associations between HLA-A^∗^03, HLA-B^∗^53 and HLA-DRB1^∗^07 and lack of protection were maintained, suggesting that these relationships are independent of each other, and could potentially exert a cumulative effect (Table 3).

**Table 3:** Independent association of HLA-A/B/DRB1 allele groups with protection in CHMI.

HLA	*n*	Odds Ratio	95% CI	*p* value
HLA-A*01	53	1.88	0.73–4.81	0.191
HLA-A*03	49	0.30	0.12–0.66	0.003
HLA-B*08	44	1.58	0.58–4.33	0.370
HLA-B*53	15	0.11	0.02–0.55	0.007
HLA-DRB1*15/*16	79	2.28	1.15–4.52	0.019
HLA-DRB1*07	56	0.36	0.17–0.76	0.007

HLA = broad serotype; *n* = number of subjects positive for allele group. Odds ratio, 95% confidence interval (CI), and *p* value refer to comparison of proportion of subjects protected between subjects positive and negative for an allele group using logistic regression and adjusting for trial, adjuvant, schedule, and other allele groups previously associated with protection or non-protection.

In contrast, the associations between HLA-A^∗^01 or HLA-B^∗^08 and RTS,S-mediated protection during CHMI were no longer significant when the presence or absence of other allele groups is taken into account (Table 3). However, if we only adjust for HLA-A^∗^03, HLA-B^∗^53, HLA-DRB1^∗^15/^∗^16 and HLA-DRB1^∗^07, these associations are maintained for both allele groups (HLA-A^∗^01, OR: 2.40, *p* = 0.027; HLA-B^∗^08, OR: 2.33, *p* = 0.046). This is consistent with the known existence of an ‘ancestral haplotype’, which is a common, highly conserved HLA haplotype, including both the HLA-A^∗^01 and HLA-B^∗^08 allele groups, known to have effects on immune responses (reviewed in [22]). Indeed, 14.9% of trial participants were positive for both HLA-A^∗^01 and HLA-B^∗^08, consistent with these allele groups being part of the most common haplotype in Caucasian Americans [23], though we cannot be certain the allele groups were on the same chromosome for all subjects and thus co-inherited. For the purposes of this analysis, this means that it is not possible to differentiate between the putative effects associated with HLA-A^∗^01 and HLA-B^∗^08, but does therefore raise intriguing suggestions regarding the impact of common haplotypes on RTS,S vaccine efficacy, as detailed in the Discussion.

### 3.3 Not all HLA allele groups associated with protection in CHMI are also associated with anti-circumsporozoite titres on day of challenge

The primary immunogenicity read-out for RTS,S clinical trials has historically been anti-CS antibody titres to the central amino acid repeat region, as these antibodies are thought to play the major RTS,S-mediated role in preventing sporozoite invasion of hepatocytes. Specifically, in Phase II trials including CHMI, the anti-CS IgG titres on the day of malaria challenge (three weeks after the last vaccine administration) are particularly of interest. We therefore sought to establish whether the associations between HLA allele groups and protection in the CHMI system were linked to a relationship between the allele groups and anti-CS titres.

To address this, the anti-CS (NANP repeat) IgG titres, available for 219 of the 222 subjects, were log transformed and compared between subjects with and without each of the six allele groups by linear regression, adjusting for trial, adjuvant, and schedule (Table 4). Of the three allele groups previously linked with protection, HLA-A^∗^01 (Coeff: 0.29; *p* = 0.047) and HLA-B^∗^08 (Coeff: 0.43; *p* = 0.005) were associated with increased anti-CS titres on the day of challenge. Conversely, a significant association was found between HLA-B^∗^53 and lower anti-CS titres (Coeff: −0.84; *p* = 0.001).

**Table 4:** Association of HLA-A/B/DRB1 allele groups with anti-CS antibody titres on day of challenge.

HLA	*n*	Coefficient	95% CI	*p* value
HLA-A*01	52	0.29	0.003–0.57	0.047
HLA-A*03	49	0.20	−0.09–0.49	0.175
HLA-B*08	43	0.43	0.13–0.73	0.005
HLA-B*53	14	−0.84	−1.33 to −0.36	0.001
HLA-DRB1*15/*16	78	0.14	−0.16–0.39	0.283
HLA-DRB1*07	56	−0.24	−0.52–0.04	0.097

HLA = broad serotype; n = number of subjects positive for allele group. Coefficient, 95% confidence interval (CI), and *p* value refer to comparison of anti-CS titres on day of challenge between subjects positive and negative for an allele group using linear regression and adjusting for trial, adjuvant, and schedule.

While these relatively low coefficients could suggest a limited relationship between HLA allele groups and anti-CS titres generated by RTS,S vaccination, it is important to recognise that the range of anti-CS titres reported in each of the trials is heavily influenced by the assay used to measure the IgG titres. While we have accounted for this by adjusting for trial in the statistical analyses, it does mean that the coefficients should not be interpreted as relating to changes in magnitude in anti-CS responses in any particular assay.

### 3.4 Associations between HLA and protection are not entirely explained by anti-circumsporozoite titres, suggesting a role for other mechanisms of HLA influence on immune responses to RTS,S

Following on from these associations between HLA allele groups with both protection/non-protection and anti-CS titres, we considered whether a relationship with anti-CS titre on day of challenge determined the relationship between allele groups and protection or lack of protection. To achieve this, we repeated the logistic regression analyses, adjusting for anti-CS titre in addition to trial, adjuvant, and schedule (Table 5). Supporting the data in Table 4 that showed no relationship between HLA-A^∗^03 or HLA-DRB1^∗^07 and anti-CS titres, controlling for anti-CS titres did not prevent our ability to detect a significant association between these allele groups and lack of protection (HLA-A^∗^03, OR: 0.26; *p* = 0.002; HLA-DRB1^∗^07, OR: 0.37, *p* = 0.014).

**Table 5:** Association of HLA-A/B/DRB1 allele groups with protection in CHMI after adjustment for anti-CS antibody titre on the day of challenge.

HLA	*n*	Odds ratio	95% CI	*p* value
HLA-A*01	52	2.02	0.89–4.56	0.091
HLA-A*03	49	0.26	0.11–0.61	0.002
HLA-B*08	43	1.72	0.72–4.09	0.223
HLA-B*53	14	0.37	0.09–1.59	0.181
HLA-DRB1*15/*16	78	1.85	0.94–3.65	0.077
HLA-DRB1*07	56	0.37	0.17–0.82	0.014

HLA = broad serotype; *n* = number of subjects positive for allele group. Odds ratio, 95% confidence interval (CI), and *p* value refer to comparison of proportion of subjects protected between subjects positive and negative for an allele group using logistic regression and adjusting for trial, adjuvant, schedule, and anti-CS titres on the day of challenge.

In contrast, while no relationship was found between HLA-DRB1^∗^15/^∗^16 and anti-CS titres at the day of challenge (Table 4), adjusting for anti-CS titre did increase the p value (*p* = 0.077, Table 5) as compared to the original analysis (*p* = 0.041, Table 2) so that the association was no longer significant at the 0.05 significance level. However, this reflects a relatively small change in Odds Ratio (OR = 1.90 in analysis without adjustment for anti-CS titres versus OR = 1.85 when adjusted for anti-CS titres) and, given the relatively small size of this exploratory analysis we avoid over-interpretation of this difference.

Finally, the three allele groups that were associated with higher (HLA-A^∗^01, HLA-B^∗^08) or lower (HLA-B^∗^53) anti-CS titres no longer had statistically significant associations with protection (HLA-A^∗^01, HLA-B^∗^08) or lack of protection (HLA-B^∗^53) when we controlled for anti-CS titres. This suggests that the mechanism linking these three allele groups and protection is related to the generation of the anti-CS antibody response.

## 4 Discussion

A total of 222 subjects from six different RTS,S Phase II trials with CHMI were included in these analyses, ten different HLA-A allele groups, 20 HLA-B allele groups (both HLA Class I), and ten HLA-DRB1 allele groups (HLA Class II) included in the analyses. We have demonstrated statistically significant associations between multiple HLA Class I and Class II allele groups and protection in the Phase II malaria challenge model: HLA-A^∗^01 and HLA-B^∗^08 were associated with protection whereas HLA-A^∗^03, HLA-B^∗^53 and HLA-DRB1^∗^07 were associated with lack of protection. A further allele group, HLA-DRB1^∗^15/^∗^16, was weakly associated with protection. These results indicate a role for HLA haplotype in determining subject responses and protective efficacy of RTS,S vaccination. Neither excluding subjects who received viral vectored vaccines in addition to RTS,S, or adjusting for adjuvant and schedule (in addition to trial) substantially affected our conclusions.

Primary analyses were performed for 37 allele groups as if a cut-off threshold was set for a minimum number of positive subjects to include in the analyses, we would be unable to detect any strong associations with less prevalent allele groups. Consequently, we acknowledge the risk of type I errors in this first set of analyses due to the multiplicity of testing; the expected false positive rate for 37 tests is 1.85 if we use the standard alpha value of 0.05 as the cut-off for determining whether a *p* value indicates a significant result (therefore expecting 5% of all tests to result in a Type I error, i.e. a false positive). While it is important to note the possibility of false positive results among the associations detected by these initial analyses, our relatively small sample size (and thus low power for a genetic association analysis) does not lend itself to use of stringent false discovery rate (FDR) control methods. Indeed, such FDR approaches are often not appropriate in genetic studies [21]). Nonetheless, the results and hypotheses generated by this exploratory analysis, discussed below, require further exploration and validation.

Firstly, it is interesting that both HLA Class I and Class II allele groups were associated with RTS,S-mediated protection in the malaria challenge model. This suggests that both CD4+ T cell (through HLA Class II peptide presentation) and CD8+ T cell (through HLA Class I peptide presentation) responses may contribute to the protective efficacy of RTS,S. While there is much data supporting a hypothetical role for CD8+ T cells in pre-erythrocytic immune responses, there has been no significant data from any RTS,S trials indicating a substantial CD8+ T cell contribution. The role of HLA Class II allele groups and CD4+ T cells is expected, through help for antibody production, though the significance remains unconfirmed. It is also possible that associations between these HLA allele groups and protection are mediated by other genes co-inherited with these allele groups due to linkage disequilibrium.

Secondly, it is intriguing that several of the allele groups associated with protection are known to be of lower frequency in sub-Saharan African populations, specifically HLA-A^∗^01 and HLA-B^∗^08 which are both part of the highly conserved A1B8DR3 ‘ancestral haplotype’ which is particularly common in Caucasians [23] and is known to have effects on immune responses (reviewed in [22]). Indeed, 25 of the study subjects from the six Phase II trials (conducted in the US and UK) included in this analysis had these three allele groups, though we cannot confirm on the same chromosome. On the other hand, HLA-B^∗^53, which is here associated with lack of protection, is known to be at a higher frequency in sub-Saharan Africans. If these HLA allele groups do influence RTS,S-mediated protection, we would potentially expect to see lower vaccine immunogenicity and efficacy in populations with a lower prevalence of ‘protective’ allele groups and a higher prevalence of ‘non-protective’ allele groups. The results presented here therefore suggest differences in HLA background may contribute to within-trial and between trial heterogeneities.

Finally, it is worth noting that the association between HLA-B^∗^53 and lack of RTS,S-mediated protection does not immediately appear to be consistent with previously published data; it has been reported that HLA-B^∗^53 is associated with protection against severe malaria in the field [24]. However, this association was dependent on the host response to a liver stage antigen (not CS protein) and furthermore it is plausible that the allele group could have no role in preventing development of blood-stage malaria, as detected by blood smear in these Phase II trials, while having a role in the prevention of development of severe malaria.

To conclude, the data presented here suggest a role for HLA genotype in RTS,S-mediated protection from malaria which is consistent with a role for both CD4+ and CD8+ T cells in protective immunity. The HLA backgrounds may explain some of the observed within and between trial heterogeneity. This work may inform further mode of action analyses and contribute to the ongoing efforts for generation of second generation highly protective malaria vaccines.

## Disclaimer

The opinions or assertions contained herein are the private views of the authors, and are not to be construed as official or as reflecting the views of the Department of the Army or the Department of Defense.
