# Supplementary material for: RTS,S malaria vaccine efficacy and immunogenicity during Plasmodium falciparum challenge is associated with HLA genotype
Source: Vaccine. 2018 Mar 14;36(12):1637–42. doi: 10.1016/j.vaccine.2018.01.069 (PMC5843576; doi:10.1016/j.vaccine.2018.01.069)
Supplement: Supplementary data 1 [file mmc1.docx]

**Supplemental Table 1. Associations of HLA-A/B/DRB1 allele groups with protection in CHMI when analyses are adjusted for trial only, or when only vaccinees receving the standard RTS,S regimen are included.**

|  | **All subjects,**  **adjusted for trial** | | | | **RRR subjects only,**  **adjusted for trial, adjuvant and schedule** | | | | | |  |  |
| --- | --- | --- | --- | --- | --- | --- | --- | --- | --- | --- | --- | --- |
| **HLA** | ***n*** | **Odds Ratio** | **95% CI** | ***p* value** | ***n*** | **Odds Ratio** | **95% CI** | | ***p* value** | |  | |
| HLA-A*01 | 53 | 2.32 | 1.15-4.69 | 0.019 | 45 | 2.39 | | 1.10-5.17 | | 0.027 | |  |
| HLA-A*03 | 49 | 0.43 | 0.21-0.87 | 0.019 | 39 | 0.53 | | 0.24-1.17 | | 0.116 | |  |
| HLA-B*08 | 44 | 2.44 | 1.14-5.21 | 0.021 | 38 | 2.35 | | 1.03-5.37 | | 0.042 | |  |
| HLA-B*53 | 15 | 0.16 | 0.04-0.62 | 0.008 | 11 | 0.15 | | 0.03-0.79 | | 0.026 | |  |
| HLA-DRB1*15/*16 | 79 | 1.94 | 1.06-3.56 | 0.032 | 63 | 1.98 | | 1.00-3.91 | | 0.050 | |  |
| HLA-DRB1*07 | 56 | 0.42 | 0.22-0.83 | 0.012 | 47 | 0.32 | | 0.14-0.70 | | 0.005 | |  |

HLA = broad serotype ; *n* = number of subjects positive for allele group. For ‘All subjects, adjusted for trial’, the odds ratio, 95% confidence interval (CI), and *p* value refer to comparison of proportion of subjects protected between subjects positive and negative for an allele group using logistic regression and adjusting for trial only. For ‘RRR subjects only, adjusted for trial, adjuvant and schedule’, the odds ratio, 95% confidence interval (CI), and *p* value refer to comparison of proportion of subjects protected between subjects positive and negative for an allele group using logistic regression and adjusting for trial adjuvant, and schedule, excluding any vaccinees who received a delayed fractional dose of RTS,S, or an ME-TRAP viral vector.
